# Study of High-Temperature Properties of Asphalt Mixtures Used for Bridge Pavement with Concrete Deck

**DOI:** 10.3390/ma14154238

**Published:** 2021-07-29

**Authors:** Piotr Pokorski, Piotr Radziszewski, Michał Sarnowski

**Affiliations:** Faculty of Civil Engineering, Warsaw University of Technology, Al. Armii Ludowej 16, 00-637 Warsaw, Poland; p.radziszewski@il.pw.edu.pl (P.R.); m.sarnowski@il.pw.edu.pl (M.S.)

**Keywords:** bridge pavement, asphalt mixtures, permanent deformation

## Abstract

The paper presents the issue of resistance to permanent deformations of bridge pavements placed upon concrete bridge decks. In Europe, bridge asphalt pavement usually consists of a wearing course and a protective layer, which are placed over the insulation (waterproofing). Protective layers of bridge pavement are commonly constructed using low air void content asphalt mixes as this provides the suitable tightness of such layers. Due to increased binder content, asphalt mixes for bridge pavement may have reduced resistance to permanent deformations. The article presents test results of resistance to permanent deformations of asphalt mixes for the protective layers. In order to determine the composition of mixtures with low air void content and resistance to permanent deformation, an experimental design was applied using a new concept of asphalt mix composition. Twenty-seven different asphalt mixture compositions were analyzed. The mixtures varied in terms of binder content, sand content and grit ratio. Resistance to permanent deformation was tested using the laboratory uniaxial cyclic compression method (dynamic load creep). On the basis of experimental results and statistical analysis, the functions of asphalt mixture permanent deformation resistance were established. This enabled a determination of suitable mixture compositions for protective layers for concrete bridge decks.

## 1. Introduction

Bridge pavement works under very specific load conditions. The bridge pavement is more exposed to rapid temperature changes and climatic factors than pavement on the earth foundation. The aggressiveness load is also higher [1,2,3,4,5]. Because the bridge pavement is much thinner than the standard road pavement, the stresses and strains are much higher [6,7,8]. There is a possibility of various types of damage. The most common types of damage are fatigue cracks on bridges with steel decks and permanent deformations on bridges with concrete decks [3,9,10,11]. The paper describes pavement on concrete decks. The pavement on the bridge should have features that will ensure durability and safety for driving vehicles [12,13,14]. An additional requirement is to ensure winter resistance to de-icing agents and water [1,9,15]. The bridge pavement asphalt layers are located on top of the insulation (waterproofing). The optimal solution is one that ensures waterproofing and resistance to permanent deformation in the protective layer. In this paper, the focus was on the protective layer due to the requirement of high tightness due to the increased content of the binder. For this reason, this layer should be subjected to a detailed analysis due to permanent deformations. There is, however, no standard methodology for designing mixtures for the protective layer. The resistance of asphalt mixtures to permanent deformation at high surface operating temperatures can be determined by many different parameters obtained in various tests [16,17]. The most popular method of evaluation of resistance to permanent deformations is examination of rut depth and increase in a single layer of the asphalt mix under a cyclical load at the temperature of 60 °C [7,18,19]. What is more, the hardness of mastic asphalt is evaluated using the static or dynamic punch penetration method. Resistance of asphalt mixes to permanent deformations is also frequently assessed using the compressive creep test at the heightened temperature of 40 °C to 60 °C. In this method, also known as ‘cyclic uniaxial compression’, cylindrical samples are subjected to a vertical compressive load that may be static or cyclic. Most often, such tests are conducted under laboratory conditions at the uniaxial load with a lateral confinement [7,20]. The compressive creep method is effective in determining the behavior of asphalt mixes with high mastic asphalt content and low air void content under cyclic loads. The article presents a new model of asphalt mix for the protective layer. In order to determine the optimal high-temperature properties and resistance to permanent deformation of the mixture, the model was tested via the cyclic compression test. This test allows for a full assessment of resistance to permanent deformation of mixtures with high waterproof and low content of air voids.

## 2. Materials and Methods

### 2.1. Materials and Mix Design

**Figure 1:**
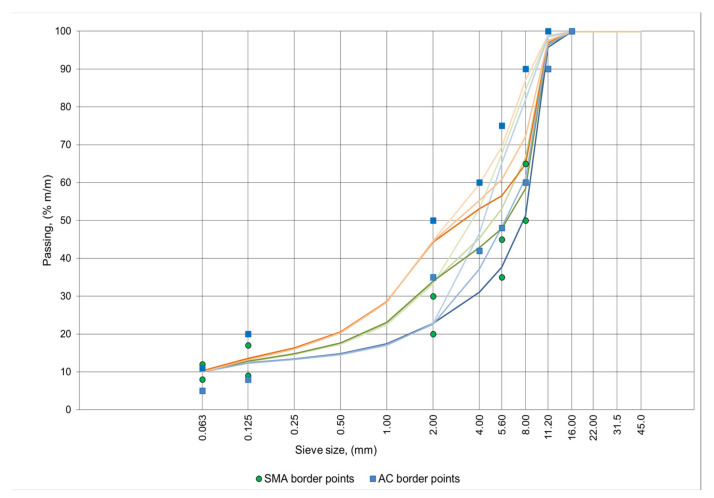
Grading curves of mineral mixtures.

The paper presents a new model of asphalt mixture. Model creation began with the design of particle size distribution curves. In the tested asphalt mixtures, basalt aggregate of various grain sizes was used, while polymer modified bitumen (PMB) 45/80-55 was employed for all mixtures (PMB 45/80-55 is a typical bitumen for these applications that is recommended in Poland). In the first stage, a set of suitable border points with particle size distribution curves were adopted. These boundary points were chosen on the basis of the Polish requirements of the Technical Guidelines WT 2-2014 [12] for the asphalt concrete (AC) mixture and the stone mastic asphalt (SMA) mixture. In the next stage, nine mineral curves were designed. These curves cover all possible compound compositions for use in the protective layer of the pavement on the bridge decks. The mineral curves are shown in Figure 1.

The mineral curves differ from each other by the amount of crushed sand content (material passing through the sieve # 2 mm) and the ratio of coarse to fine grit. The same amount of filler fraction was assumed in all mixtures at the level of 10%. The value of 10% was set in order to ensure adequately high tightness of all mixtures. The lower particle size distribution curves presented in Figure 1 correspond to mixtures with a particle size close to mastic–grit mixture. The upper graining curves correspond to mixtures of asphalt concrete with an increased content of sand fraction.

The binder content was assumed on the basis of the binder film thickness analysis for typical asphalt mixtures used for the protective layer of the surface of bridge pavement structures.

The following variables were thus adopted for the construction of the asphalt mix model:

- Amount of mineral material crushed sand 0.063/2 (three levels: 12%, 23%, 34%).
- Ratio of coarse 8/11 to fine grit 2/8 (three levels: 0.3; 1.0; 1.7).
- Binder content (three levels: 5%; 6%; 7%).

The adopted levels of individual variables result from the analysis of the compositions of mineral–asphalt mixtures used in Poland for the protective layer of bridge decks.

### 2.2. Testing Methods

The asphalt mixes were compacted using a gyratory compactor in accordance with PN-EN 12697-31 in cylindrical steel molds with a diameter of 150 mm and a height of 60 mm. For all mixes, the gyratory number was 60 and the compaction temperature was 145 °C. Five samples were made for each type of mix. The low air void content was achieved by adjusting the bitumen content. This allowed for the obtaining of samples of asphalt mixes with a developed mastic structure and high binder content that are typical for highly leak-tight mixes, and which were subsequently subjected to tests of resistance to permanent deformations.

The air void content was calculated from density and bulk density according to PN-EN 12697-8.

In order to evaluate the resistance of the asphalt mixes to permanent deformations, the test plan included the cyclic uniaxial compression (dynamic load creep) method according to PN-EN 12697-25. This method effectively characterizes the high-temperature properties of mixes with reduced air void content [20].

**Figure 2:**
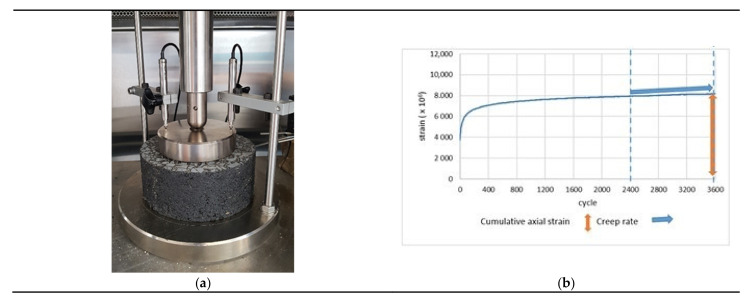
(**a**) The test apparatus of the cyclic uniaxial compression method; (**b**) the test result graph of the uniaxial cyclic compression method as applied to one sample.

Testing of resistance of asphalt mixes to permanent deformations using the cyclic uniaxial compression method consists of repeatedly applying upon the sample a stress load of the same value. The test is conducted at the temperature of 52 °C and it lasts for 3600 cycles. Rectangular pulse loading was applied. The conducted test covered five samples of each type of the asphalt mixture. Figure 2a presents the test apparatus with sample. Figure 2b presents a single test result graph.

The results of the uniaxial cyclic compression test are the cumulative axial strain and the creep rate.

Here, the cumulative axial strain is:

(1) $$\varepsilon_{n}=\left(\frac{u_{n}}{t_{i}}\right)$$

where:

$\varepsilon_{n}$ is the cumulative axial strain of the test specimen after n loading cycles,
$u_{n}$ is the cumulative permanent deformation of the test specimen after n loading cycles,
$t_{i}$ is the initial thickness of the specimen.

The creep rate is:

(2) $$f_{c}=\frac{\varepsilon_{n1}-\varepsilon_{n2}}{n_{1}-n_{2}}\times 10,000$$

where

$f_{c}$ is the creep rate,
$\varepsilon_{n1}$; $\varepsilon_{n2}$ is the cumulative axial strain of the test specimen after $n_{1}$; $n_{2}$ loading cycles,
$n_{1}$; $n_{2}$ are the repetitive loading cycles.

In the article, the creep rate ($f_{c}$) was adopted for the deformation levels $\varepsilon_{n1}$; $\varepsilon_{n2}$ according to [20] equal to $n_{1}$ = 3600 cycles over $n_{2}$ = 2400. Cumulative axial strain was calculated for a final cycle of 3600 cycles.

## 3. Results

### 3.1. Results of Permanent Deformation Resistance Test

A summary of all the results of resistance to permanent deformation and the levels of variables in individual mixtures are presented in Table 1.

**Figure 3:**
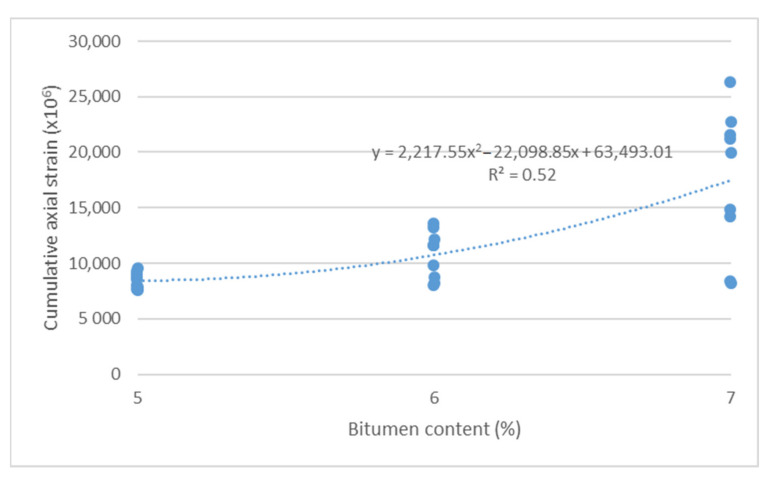
Cumulative axial strain vs. bitumen content.

**Figure 4:**
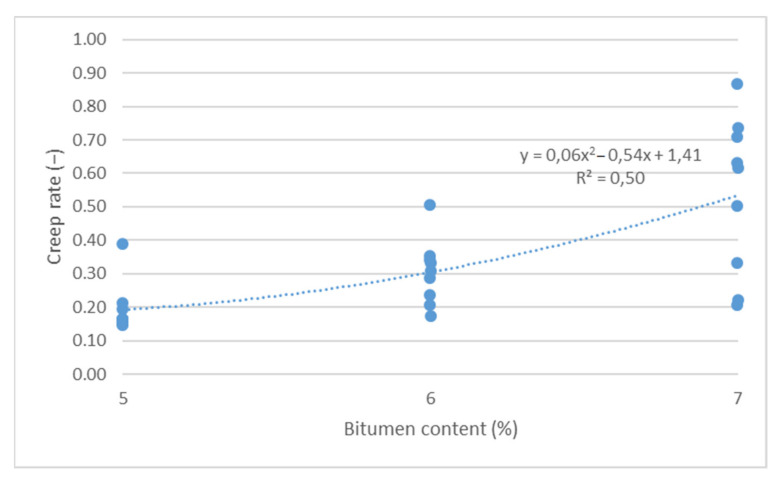
Creep rate vs. bitumen content.

**Figure 5:**
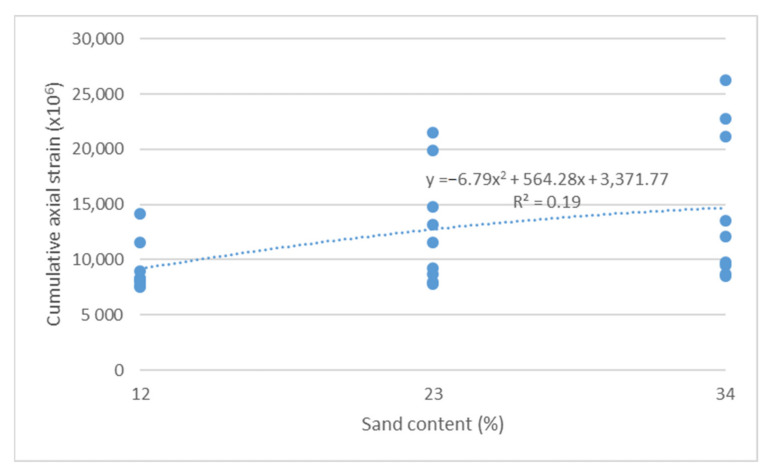
Cumulative axial strain vs. sand content.

**Figure 6:**
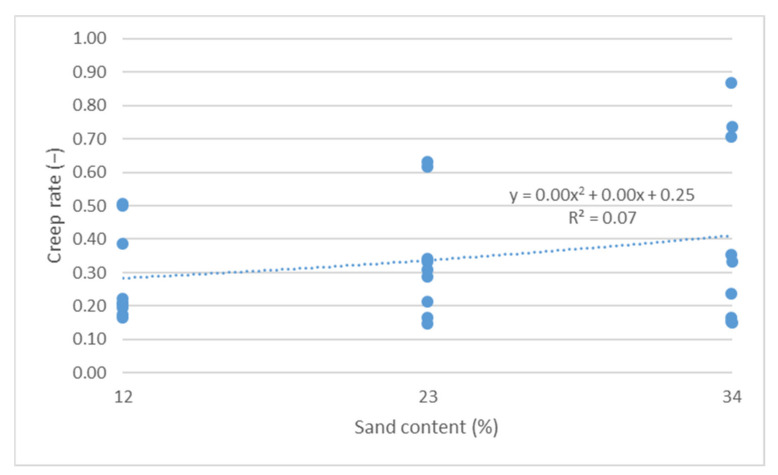
Creep rate vs. sand content.

**Figure 7:**
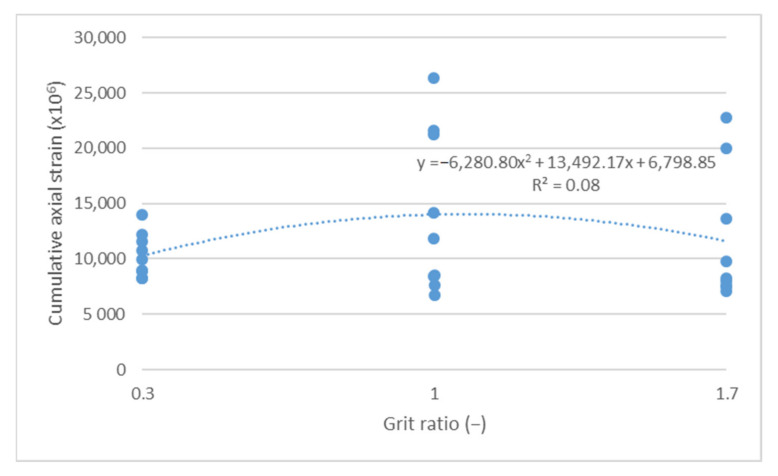
Cumulative axial strain vs. grit ratio.

**Figure 8:**
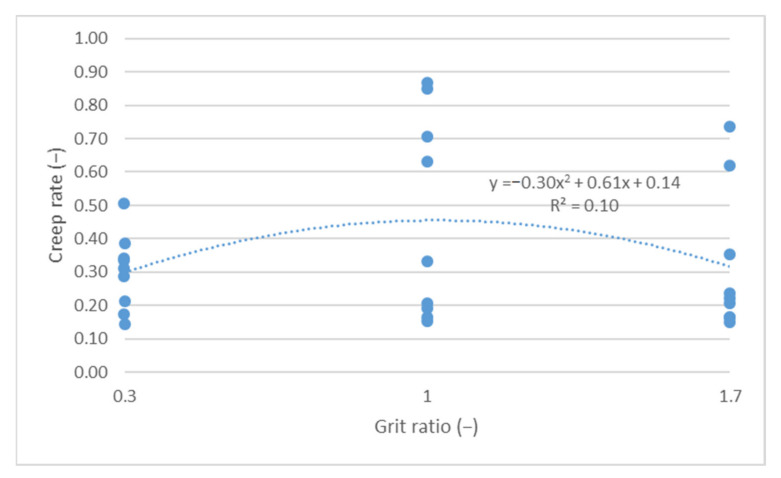
Creep rate vs. grit ratio.

The cumulative axial strain and creep rate test results, as per the research program, are shown as a function of the following parameters describing the asphalt mixture: asphalt content (Figure 3 and Figure 4), sand content (Figure 5 and Figure 6) and grit ratio (Figure 7 and Figure 8).

Figure 3 and Figure 4 indicate that increasing asphalt content in the mix adversely impacts the resistance of asphalt mixes for bridge pavement to permanent deformations, as increasing asphalt content increases both the cumulative axial strain and the creep rate. These dependencies can be described by a second order polynomial with a good degree of match R^2^ > 0.5.

Figure 5 and Figure 6 indicate that increasing sand content in mixes also adversely impacts the resistance of asphalt mixes to permanent deformations. As demonstrated, a higher sand content leads to increased values of both parameters describing the resistance of mixes to permanent deformations.

Figure 7 and Figure 8 indicate that a higher content of grit with high grain size does not significantly impact the parameters describing resistance to permanent deformations. The values of both the cumulative axial strain and creep rate, despite a significant increase in the grit ratio coefficient—from 0.3 to 1.7—remains at a relatively similar level.

The authors conducted a correlation analysis covering the test results (r correlation factor assessment) that exhibited high dependence of the cumulative axial strain on bitumen content (r = 0.69), average on sand content (r = 0.42) and poor on grit ratio (r = 0.03). Creep rate test results also confirmed a high correlation between binder content in the mix (r = 0.68), while the correlation with sand content and grit ratio content was poor. No correlation was found between the grit ratio and the cumulative axial strain and creep rate. The results are presented in Table 2.

### 3.2. Test Results for Air Void Content in an Asphalt Mix

**Figure 9:**
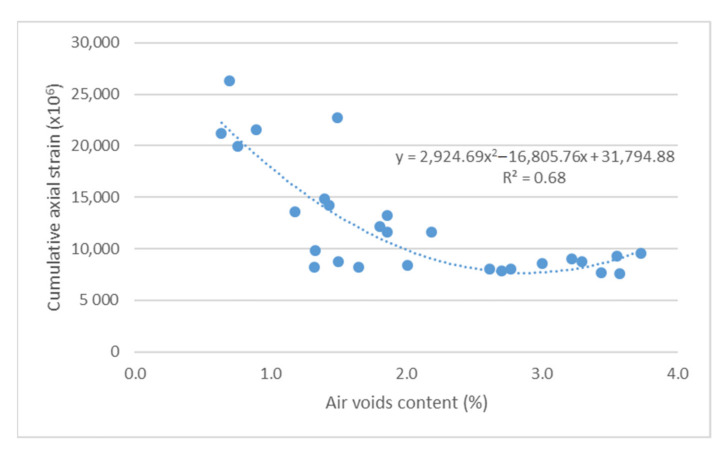
Cumulative axial strain vs. air void content.

**Figure 10:**
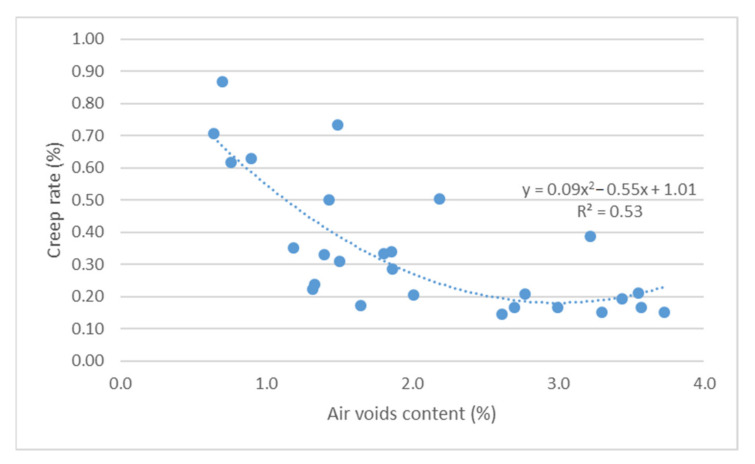
Creep rate vs. air void content.

The impact of air void content on the resistance of asphalt mixes to permanent deformations was conducted using two parameters: cumulative axial strain and creep rate (Figure 9 and Figure 10).

Based on the results shown in Figure 9 and Figure 10, it can be concluded that there is a relationship between the parameters for assessing the resistance of bridge pavement mixes to permanent deformations, and the content of air void. Increased air void content leads to increased resistance of the mix to permanent deformations, as per a second-degree function (R^2^ > 0.5 correlation power). The conducted correlation analysis confirmed the existence of a very high correlation between the results of air void content and the adopted parameters for assessing the resistance to permanent deformations (r > 0.7).

Based on the correlation analysis, it was concluded that there was a very high correlation dependency between the air void content and binder content (r = 0.88). The sand content and grit ratio parameter turned out to be insignificant due to correlation dependencies.

**Figure 11:**
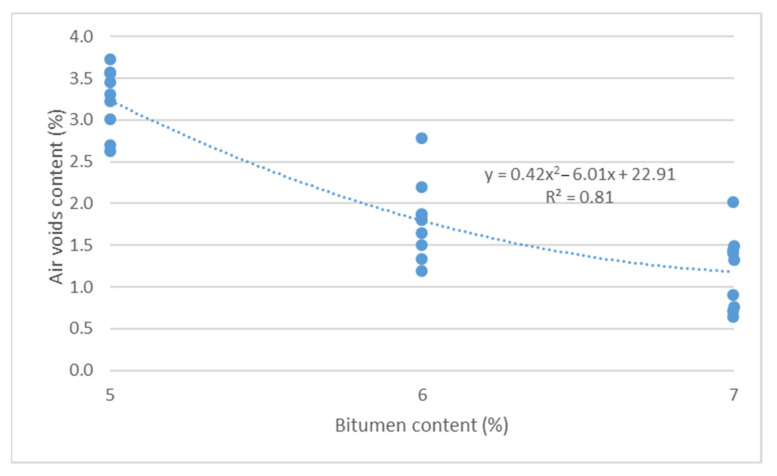
Air void content vs. bitumen content.

**Figure 12:**
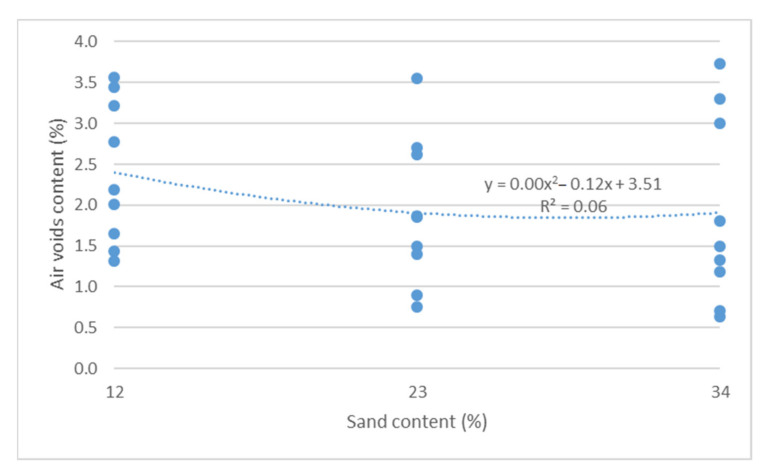
Air void content vs. crushed sand content.

Air void content test results as a function of binder content and sand content are shown in Figure 11 and Figure 12.

Based on the results from Figure 11 and Figure 12, it should be concluded that increased bitumen content and sand content lead to reduced air void content, in accordance with a second-degree function. There is a high degree of matching of a second-degree function between air void content and bitumen content (R^2^ = 0.81).

An analysis of variance and regression was conducted. The analysis results are shown in Table 3.

The results from Table 3 indicate that binder content and sand content very significantly impact the air void content in mixes.

Based on the analysis of many forms of equations, the one with the highest value of R_2_ = 0.87 was selected. The following equation was obtained:

(3) $$V_{a}=24.34438-6.01182x_{1}+0.41548x_{1}^{2}-0.11699x_{2}+0.00206x_{2}^{2}$$

where:

$x_{1}$ is binder content,

$x_{2}$ is sand content.

This equation applies to asphalt mixtures with an asphalt content of 5–7% and a sand content of 12–34%.

### 3.3. Test Result Variation Analysis

The authors conducted a variance analysis—main effect analysis, which determined the significance of the impact of individual factors (binder content, sand fraction content and grit ratio) on air void content and resistance to permanent deformations—cumulative axial strain and creep rate. Table 4 shows detailed variance analysis results.

Based on the results in Table 4, it should be concluded that the parameters describing resistance to permanent deformations (cumulative axial strain and creep rate) are most impacted by binder content. The sand content is also important in the event of a cumulative axial strain.

### 3.4. Functional Relationships for Describing the Resistance of Asphalt Mixes to Permanent Deformations by Cyclic Compression Test

A cumulative axial strain regression model, creep rate inclination model and an air void content regression model as a function of assessment parameters for the asphalt mix composition were developed to describe the resistance of bridge structure protective layer asphalt mixes to permanent deformations.

Based on regression analysis from the test results, R^2^ correlation power value maximization was used to determine a model in the form:

(4) $$Y\left\{y_{1},y_{2}\right\}=a_{0}+a_{1}x_{1}+a_{2}x_{1}^{2}+a_{3}x_{2}+a_{4}x_{2}^{2}+a_{5}x_{1}x_{2}$$

where

$y_{1}$ is cumulative axial strain,
$y_{2}$ is creep rate.

The adopted independent variables were the asphalt mix composition evaluation parameters that significantly impact the y_1_, y_2_ values:

$x_{1}$ is binder content,
$x_{2}$ is sand fraction content.

The regression analysis results are shown in Table 5.

The level of significance in the analysis was *p* < 0.10. From the results in Table 5, it was found that the binder content and sand content have a significant impact on cumulative axial strain. The binder content also has a significant impact on creep rate.

The following equations were obtained based on regression analysis after reducing non-significant components of polynomials:

(5) $$y_{1}=84,204.6-24,622.8x_{1}+1839.4x_{1}^{2}-1343.4x_{2}+309.0x_{1}x_{2}$$

(6) $$y_{2}=-0.784966+0.190305x_{1}$$

The developed regression function describes the sought end parameters well. Model matching coefficients are R^2^ = 0.82 (for $y_{1}$), R^2^ = 0.78 (for $y_{2}$).

Equation (5) is used to evaluate the cumulative axial strain with a binder content within 5–7% and sand content within the range of 12–34%. Equation (6) is used to evaluate the creep ratio with a binder with a content of 5–7% and sand content in the range of 12–34%.

Table 6 presents the results of cumulative axial strain and creep ratio calculations according to Equations (5) and (6) for selected mixtures used for the protective layer on bridge decks in Poland.

Based on the results of the calculations presented in Table 6, it can be concluded that the equations created in the article allow us to determine the resistance to permanent deformation of asphalt mixtures used for the protective layer of the bridge decks.

The next study stage will involve subjecting the obtained equations to validation using mixes sampled from bridge structure pavements under high traffic loads at different operating periods that are characterized by confirmed high resistance to permanent deformations. The axial strain, creep rate and air void content values will be determined, taking into account the mixes applied to bridge structures that were implemented within the last 20 years that are characterized by high exploitation durability. The obtained equations and limit values of the parameters will enable the development of a composition of bridge structure asphalt mix that is resistant to permanent deformations.

The asphalt mix composition determination procedure will be as follows:

- Predetermining the asphalt mix composition based on applicable technical requirements.
- Assessing the resistance of the adopted mix to permanent deformation according to the obtained equations, namely, the cumulative axial strain model and creep rate model, with composition correction—taking into account the limit values for different traffic load categories.
- Assessing the air void content in the designed asphalt mix, together with composition correction—taking into account the limit value for air void content.
- Final assessment of mix resistance to permanent deformation as per developed models.

## 4. Conclusions

The following conclusions can be drawn based on the state-of-the-art, laboratory test results and analysis:

- Asphalt bridge pavement is subjected to specific working conditions relative to pavements located on the ground and requires a different approach to modeling the structural layer asphalt mix composition—the protective layer in particular.
- The state of the art shows that designing the composition of mixtures for protective layers does not take into account the specific working conditions and the function that this layer must perform in the bridge pavement.
- For the protective layers, compactable mineral-asphalt mixtures with a grain size corresponding to the SMA and AC mixtures with an increased binder content are commonly used.
- Asphalt pavement on bridge structures with a concrete deck are particularly exposed to the formation of damage in the form of permanent deformations or ruts.
- The required high waterproofing of bridge pavements achieved through low air void content and increasing protective layer binder content additionally affects the susceptibility of such pavements to the formation of strains, especially under high operating temperatures.
- It is possible to model the high-temperature properties of bridge structure protective layer mixes based on results of cyclic uniaxial compression test.
- The worked-out functional relationships enable the assessment of the resistance to permanent deformations of bridge pavement protective layer asphalt mixes.
- A second-degree functional relationship shall evaluate a protective layer asphalt mix composition in terms of ensuring low air void content (high waterproofing asphalt mixture).
- The developed functional equations enable the selection of asphalt mix composition with high resistance to permanent deformations.
- The developed models make it possible to differentiate the composition of the mixtures in terms of their resistance to permanent deformation in accordance with the cyclic compression test. The models enable the selection of the best parameters of resistance to permanent deformation, taking into account the air void content.

## Figures and Tables

**Table 1:** Levels of variables in individual mixtures and their results of permanent deformation resistance test.

Mixture Code	Binder Content (%)	Sand Content (%)	Grit Ratio (-)	Cumulative Axial Strain (×10^6^)	Creep Rate (-)	Air Void Content (%)
1A	5	12	1.7	7544	0.17	3.6
1B	6	12	1.7	8167	0.17	1.6
1C	7	12	1.7	8231	0.22	1.3
2A	5	34	0.3	9508	0.15	3.7
2B	6	34	0.3	12,124	0.33	1.8
2C	7	34	0.3	22,736	0.74	1.5
3A	5	23	1	7842	0.17	2.7
3B	6	23	1	8733	0.31	1.5
3C	7	23	1	19,942	0.62	0.8
4A	5	23	0.3	9262	0.21	3.6
4B	6	23	0.3	11,600	0.29	1.9
4C	7	23	0.3	14,754	0.33	1.4
5A	5	12	0.3	8964	0.39	3.2
5B	6	12	0.3	11,526	0.50	2.2
5C	7	12	0.3	14,150	0.50	1.4
6A	5	23	1.7	7967	0.14	2.6
6B	6	23	1.7	13,185	0.34	1.9
6C	7	23	1.7	21,537	0.63	0.9
7A	5	34	1.7	8542	0.16	3.0
7B	6	34	1.7	13,557	0.35	1.2
7C	7	34	1.7	26,278	0.87	0.7
8A	5	12	1	7591	0.19	3.4
8B	6	12	1	7963	0.21	2.8
8C	7	12	1	8352	0.21	2.0
9A	5	34	1	8718	0.15	3.3
9B	6	34	1	9733	0.24	1.3
9C	7	34	1	21,171	0.71	0.6

**Table 2:** Correlation analysis results.

Dependent Variable	Factors								
	Binder Content			Sand Content			Grit Ratio		
	r	Correlation Level	Significance	r	Correlation Level	Significance	r	Correlation Level	Significance
Cumulative axial strain	0.69	high	*p* < 0.05	0.42	medium	*p* < 0.05	0.03	low	*p* > 0.05
Creep rate	0.68	high	*p* < 0.05	0.25	low	*p* > 0.05	0.08	low	*p* > 0.05

**Table 3:** Variance analysis results.

Dependent Variable	Factors					
	Binder Content		Sand Content		Grit Ratio	
	Significance (p)	Significance Level	Significance (p)	Significance Level	Significance (p)	Significance Level
Air void content	0.00	very significant	0.00	very significant	0.05	low significance

**Table 4:** Variance analysis results.

Dependent Variable	Factors					
	Binder Content		Sand Content		Grit Ratio	
	Significance (p)	Significance Level	Significance (p)	Significance Level	Significance (p)	Significance Level
Cumulative axial strain	0.00	very significant	0.02	significant	0.48	insignificant
Creep rate	0.00	very significant	0.49	insignificant	0.36	insignificant

**Table 5:** Regression analysis results.

Equation Component	Cumulative Axial Strain	Significance (p)	Creep Rate	Significance (p)
$a_{0}$	84,204.6	0.043	−0.784966	0.002
$a_{1}$—binder content	−24,622.8	0.069	0.190305	0.000
$a_{2}$—binder content^2^	1839.4	0.098		
$a_{3}$—sand content	−1343.4	0.030		
$a_{4}$—sand content^2^	−6.1	0.498		
$a_{5}$—binder content × sand content	309.0	0.000		

**Table 6:** Calculation results for selected mixtures.

Mixture Type	Binder Content	Sand Content	Air Void Content (Calculated)	Cumulative Axial Strain (Calculated)	Creep Rate (Calculated)
PL1–SMA	5.9	15	2.4	10,151	0.34
PL2–SMA	6.5	16	1.8	11,844	0.45
PL3–AC	5.0	26	3.3	12,313	0.16

## Data Availability

The data is available within the article and can be requested from the corresponding authors.
